# Engineering of *Yarrowia lipolytica* for terpenoid production

**DOI:** 10.1016/j.mec.2022.e00213

**Published:** 2022-11-07

**Authors:** Jonathan Asmund Arnesen, Irina Borodina

**Affiliations:** The Novo Nordisk Foundation Center for Biosustainability, Technical University of Denmark, Kemitorvet 220, 2800, Kgs. Lyngby, Denmark

## Abstract

Terpenoids are a group of chemicals of great importance for human health and prosperity. Terpenoids can be used for human and animal nutrition, treating diseases, enhancing agricultural output, biofuels, fragrances, cosmetics, and flavouring. However, due to the rapid depletion of global natural resources and manufacturing practices relying on unsustainable petrochemical synthesis, there is a need for economic alternatives to supply the world's demand for these essential chemicals. Microbial biosynthesis offers the means to develop scalable and sustainable bioprocesses for terpenoid production. In particular, the non-conventional yeast *Yarrowia lipolytica* demonstrates excellent potential as a chassis for terpenoid production due to its amenability to industrial production scale-up, genetic engineering, and high accumulation of terpenoid precursors. This review aims to illustrate the scientific progress in developing *Y. lipolytica* terpenoid cell factories, focusing on metabolic engineering approaches for strain improvement and cultivation optimization.

## Highlights

• *Yarrowia lipolytica* is an emerging host for terpenoid production.
• Mevalonate pathway engineering is widely used and effective for terpenoid production.
• Modulating lipid storage and acetyl-CoA abundance can increase terpenoid production.
• Compartmentalization, morphology, and enzyme engineering are promising strategies.
• A wide range of substrates can be used for terpenoid production by *Y. lipolytica*.

## 1 Introduction

Terpenoids or isoprenoids represent a vast and structurally diverse class of small molecules involved in specialized and general metabolism across the entire kingdom of life (Ashour et al., 2010; Gershenzon and Dudareva, 2007). A unifying feature for all terpenoids is their genesis from 5 carbon (C_5_) units. The classification of terpenoids is based on the number of C_5_-units comprising the basic scaffolds: hemiterpenes (C_5_), monoterpenes (C_10_), sesquiterpenes (C_15_), diterpenes (C_20_), sesterterpenes (C_25_), triterpenes (C_30_), sesquarterpenes (C_35_), and tetraterpenes or carotenoids (C_40_) (Ashour et al., 2010; Sato, 2013). In addition, the isoprene scaffolds may undergo re-arrangement and other modifications, creating numerous diverse structures. Many terpenoids hold value as potent pharma- and nutraceuticals, biofuels, or as flavor, colour, or cosmetic agents (Tetali, 2018). But harvesting terpenoids from natural resources or manufacturing by chemical synthesis is often economically disadvantageous or unsustainable (Idris and Mohd Nadzir, 2021; Pateraki et al., 2017). Therefore, considerable attention has been applied to the producing terpenoids via engineered microbes. The common chassis organisms are *Escherichia coli* and *Saccharomyces cerevisiae* since they are easy to cultivate and engineer, and much is known about their biology (Moser and Pichler, 2019). Recently, the oleaginous yeast *Yarrowia lipolytica* has been intensely researched for its terpenoid production capabilities. This yeast offers benefits like broad substrate utilization, sequenced genomes, and available genetic engineering toolkits, with several strains having achieved GRAS-status (Darvishi et al., 2018; Dujon et al., 2004; Holkenbrink et al., 2018; Magnan et al., 2016; Turck et al., 2019). This review aims to showcase both established and nascent strategies for improving terpenoid production in *Y. lipolytica*.

## 2 Terpenoid biosynthesis in *Y. lipolytica*

**Fig. 1:**
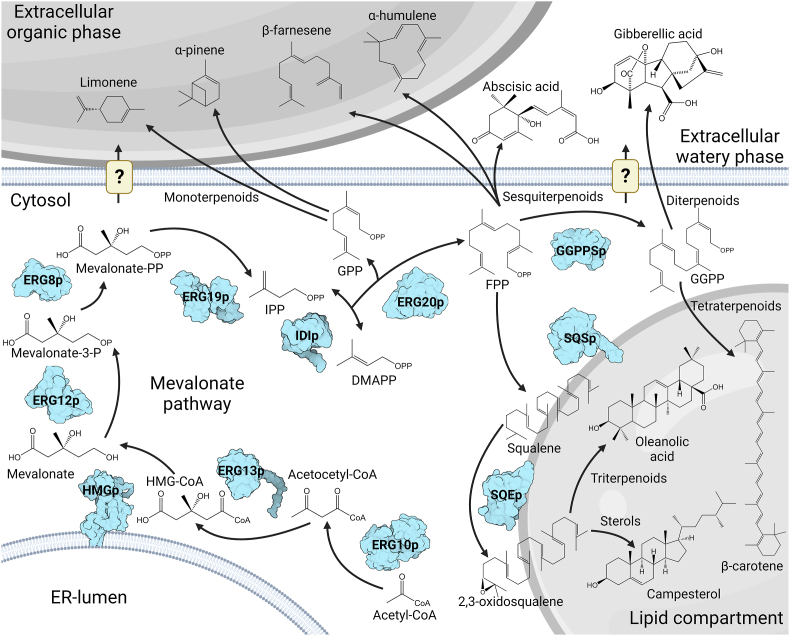
The mevalonate pathway and examples of terpenoids produced by engineered *Yarrowia lipolytica* strains. The mevalonate pathway occurs in the yeast cytosol. Many terpenoids accumulate either in lipid compartments or the extracellular matrix, although the terpenoid transport mechanisms in *Y. lipolytica* remain poorly understood. Metabolite abbreviations: CoA, co-enzyme A. HMG-CoA, 3-hydroxy-3-methylglutaryl-coenzyme A. P, phosphate. PP, diphosphate. IPP, isopentenyl diphosphate. DMAPP, dimethylallyl diphosphate. GPP, geranyl diphosphate. FPP, farnesyl disphosphate. GGPP, geranylgeranyl diphosphate. Enzyme abbreviations: ERG10p, acetyl-CoA acetyltransferase. ERG13p, 3-hydroxy-3-methylglutaryl-CoA synthase. HMGp, 3-hydroxy-3-methylglutaryl-CoA reductase. ERG12p, mevalonate kinase. ERG8p, phosphomevalonate kinase. ERG19p, mevalonate diphosphate decarboxylase. IDIp, isopentenyl diphosphate isomerase. ERG20p, geranyl, and farnesyl diphosphate synthase. GGPPSp, geranylgeranyl diphosphate synthase. SQSp, squalene synthase. SQEp, squalene epoxidase. The depicted protein structures are of *Saccharomyces cerevisiae* homologs. The ERG10p (PDB ID: 5XYJ), ERG19p (PDB ID: 1FI4), and GGPPS (PDB ID: 2E90) structures are based on X-ray crystallography (Berman et al., 2000; Bonanno et al., 2001; Guo et al., 2007; Zhou, 2018). The ERG13p, HMGp, ERG12p, ERG8p, IDIp, ERG20p, SQSp, and SQEp structures are based on AlphaFold predictions (Jumper et al., 2021; Varadi et al., 2022).

Terpenoid biosynthesis in *Y. lipolytica* occurs via the cytosolic mevalonate (MVA) pathway starting from the condensation of two acetyl-CoA units into acetoacetyl-CoA catalyzed by the acetyl-CoA acetyltransferase (ERG10p) (Fig. 1) (Cao et al., 2016; Ma et al., 2019; Miziorko, 2011). The 3-hydroxy-3-methylglutaryl-CoA synthase (ERG13p) condensates acetoacetyl-CoA and another acetyl-CoA unit forming 3-hydroxy-3-methylglutaryl-CoA (HMG-CoA), which is then reduced by the 3-hydroxy-3-methylglutaryl-CoA reductase (HMGp) into mevalonate. Subsequently, mevalonate is phosphorylated by the mevalonate kinase (ERG12p) and the phosphomevalonate kinase (ERG8p), forming mevalonate-5-diphosphate, which in turn is decarboxylated by the mevalonate diphosphate decarboxylase (ERG19p) into isopentenyl diphosphate (IPP). The isopentenyl diphosphate isomerase (IDIp) can reversibly convert IPP to its isomer dimethylallyl diphosphate (DMAPP). Further enzymatic condensation of IPP and DMAPP results in the formation of phosphorylated isoprene units such as C_10_ geranyl diphosphate (GPP), C_15_ farnesyl diphosphate (FPP), and C_20_ geranylgeranyl diphosphate (GGPP) that serve as precursors for other terpenoids. Interestingly, a recent report suggested the presence of the methylerythritol phosphate (MEP) pathway in *Y. lipolytica* based on liquid chromatography mass-spectrometry and ^13^C-analysis of *Y. lipolytica* metabolites from various cultivation conditions, although the MEP-pathway commonly occurs in plant plastids, bacteria, and algae (Dissook et al., 2021; Rohmer, 1999). Yet still, no enzymes have been reported to be involved in the hypothetical *Y. lipolytica* MEP-pathway. Various terpenoids have been produced in *Y. lipolytica* with many examples for mono-, sesqui-, tri-, and tetraterpenoids, and a few instances of di- and hemiterpenoids and apocarotenoids (Table 1). This includes flavor and fragrance additives such as the monoterpenoids limonene, α-pinene, and linalool, and the sesquiterpenoids valencene, (+)-nootkatone (Cao et al., 2017; Cheng et al., 2019; Guo et al., 2018; Wei et al., 2021b). Likewise, the sesquiterpene biofuel candidates α- and β-farnesene have been produced at 25.6 and 22.8 g/L, respectively, while the potential pharmaceutical sesquiterpene α-humulene was produced at 3.2 g/L (Guo et al., 2021; Y. Liu et al., 2019; T. Shi et al., 2021). *Y. lipolytica* has also been engineered to produce medicinal triterpenoids like oleanolic acid, protopanaxadiol, ginsenoside K, and the cosmetic ingredient squalene (Gao et al., 2017b; D. Li et al., 2020; Li et al., 2019; Wu et al., 2019). Even plant hormones like the sesquiterpenoid abscisic acid and gibberellin diterpenoids (GAs) useable for agriculture have been produced in this yeast (Arnesen et al., 2022; Kildegaard et al., 2021). While *Y. lipolytica* shows general potential for terpenoid production, its carotenoid production capabilities are perhaps the most striking. Indeed, the cultivation of an engineered *Y. lipolytica* strain recently resulted in 39.5 g/L and 494 mg/g DCW β-carotene. These production metrics exceed what has been reported in scientific and patent literature on β-carotene production by recombinant and native microbes (Costa Perez et al., 2017; M. Liu et al., 2021).

**Table 1:** Overview of metabolic engineering strategies for terpenoid production in *Y. lipolytica*.

Compound	Carbon Source	Parental Strain	Modifications related to terpenoid biosynthesis	Titer	Reference
**Hemiterpenoids:**					
**Isoprene**	glucose	Po1g	↑HMG ↑ERG13 ↑IDI ↑PmISPS	530.4 μg/L (sealed vials)	Shaikh and Odaneth (2021)
**Monoterpenoids:**					
**Limonene**	Glucose Pyruvic acid	Po1f	*↑TArLS ↑tSlNDPS1 ↑HMG ↑ERG12*	23.6 mg/L (shakeflask)	Cao et al. (2016)
**Limonene**	Glucose	Po1g	*↑HMG (↑ClLS or ↑MsLS)*	D-limonene: 11.7 mg/L or	Pang et al. (2019)
				L-limonene: 11.1 mg/L, respectively (bioreactor)	
**Limonene**	Glycerol Citrate	Po1f	*↑TArLS ↑tSlNDPS1 ↑HMG ↑ERG12*	165.3 mg/L (bioreactor)	Cheng et al. (2019)
**Limonene**	Glucose	ATCC 20460	*↑HMG ↑ERG12 ↑ACL1 ↑SeACS*^*L641P*^*↑IDI ↑ERG20*^*F88W*^*–*^*N119W*^*↓SQS ↑PfLS*	35.9 mg/L (glass tube)	Arnesen et al. (2020)
**Limonene**	Lignocellulosic hydrolysate Citric acid	Po1f	*↑ssXR ↑ssXDH ↑XKS*	20.57 mg/L (shake flasks)	Yao et al. (2020)
			*↑TArLS ↑SltNDPS1 ↑HMG ↑ERG12*		
**Limonene**	Waste cooking oil	Po1f	*(↑ClLS or ↑MsLS) ↑HMG ↑IDI ↑tSlNDPS1*	91.24 (D-limonene) and 83.06 (L-limonene) mg/L (shake flasks)	Li et al. (2022)
**Linalool**	Citrate Pyruvate	Po1f	*↑AaLIS ↑ERG20*^*F88W*^*–*^*N119W*^*↑HMG ↑IDI*	6.96 mg/L (shake flask)	Cao et al. (2017)
**α-pinene**	Waste cooking oil, soybean oil, or lignocellulosic hydrolysate medium	Po1f	*↑HMG ↑tSlNDPS1 ↑tPtPS ↑ERG8,12 ↑MBP-ERG12 ↑AMPD*	33.8, 36.6 or 36.1 mg/L for each carbon source, respectively (shake flasks)	Wei et al. (2021b)
**Sesquiterpenoids:**					
**Abscisic acid**	Glucose	ATCC 20460	*↑HMG ↑ERG12 ↑ACL1 ↑SeACS*^*L641P*^*↑IDI ↑ERG20 ↓SQS ↑BcABA1-4 ↑BcCPR1 ↑POS5 ↑AtDTX50*	263.5 mg/L (deepwell plate)	Arnesen et al. (2022)
**Amorphadiene**	Glucose	Po1g	*↑AaADS ↑HMG ↑ERG12*	171.5 mg/L (shakeflask)	Marsafari and Xu (2020)
**α-bisabolene**	Waste cooking oil	Po1g	*↑HMG ↑GcABCG1* (*↑AgαBS, ↑ZoβBS, or ↑HaγBS*)	973.1 mg/L,	Zhao et al. (2021)
**β-bisabolene**				68.2 mg/L, or	
**γ-bisabolene**				20.2 mg/L, respectively (culture tubes)	
**(−)-α-bisabolol**	Glucose	Po1f	*↑POT1 ↑MrBBS ↑tHMG ↑ERG20 ↓SQS*	364.23 mg/L (shake flasks)	(Yirong Ma et al., 2021)
**α-farnesene**	Glucose Fructose	Po1h	*↑tScHMG ↑IDI ↑MdFS-L-ERG20*	260 mg/L (bioreactor)	Yang et al. (2016)
**α-farnesene**	Glucose	Po1f	*↑EcAtoB ↑BpHMG ↑ERG13 ↑MdFS-L-ERG20 ↑ERG12 ↑IDI ↑ERG8,19 ↑GPPS*	25.55 g/L (bioreactor)	(Y. Liu et al., 2019)
**α-farnesene**	Glucose Glycerol	Po1f	*↑FS-L-ERG20 ↑tScHMG1 ↑IDI ↑HMG ↑ERG19*	2.57 g/L (bioreactor)	(S. C. Liu et al., 2020)
**α-farnesene**	Oleic acid	Po1f	*↑VHb ↑MdFS-L-ERG20 ↑ERG12 ↑IDI ↑ERG8,19*	10.2 g/L (bioreactor)	(Y. Liu et al., 2021)
**β-farnesene**	Glucose	ATCC 20460	*↑HMG ↑ERG12 ↑ACL1 ↑SeACS*^*L641P*^*↑IDI ↑ERG20 ↑AaBFS*	955 mg/L (glass tube)	Arnesen et al. (2020)
**β-farnesene**	Glucose	Po1f	*ΔDGA1 ΔDGA2 ↑tHMG ↑BFS ↑ERG8,10,12, 13,19,20 IDI Δgut2 Δpox3,4,5,6*	22.8 g/L (bioreactor)	(T. Shi et al., 2021)
**α-humulene**	Glucose	Po1f	*↑POT1 ↑AcACHS2*_*PTS*_*↑RpHMG*_*PTS*_*↑ANT1 ↑(ERG12,8,20,10,13,19)*_*PTS*_*↑IDI*_*PTS*_	3.2 g/L (bioreactor)	Guo et al. (2021)
**α-santalene**	Glucose	ATCC 201249	*↑ClSTS ↑ERG8 ↑tHMG*	27.92 mg/L (bioreactor)	Jia et al. (2019)
**Nootkatone**	Glucose	ATCC 201249	*↑CnVS ↑CnCYP706M1-tAtATR1 ↑tScHMG ↑ERG20*	Nootkatone: 978.2 μg/L	Guo et al. (2018)
**Valencene**				Valencene: 22.8 mg/L (shake flask)	
**Valencene**	Glucose	ATCC 20460	*↑HMG ↑ERG12 ↑ACL1 ↑SeACS*^*L641P*^*↑IDI ↑ERG20 ↓SQS ↑CnVS*	113.9 mg/L (glass tube)	Arnesen et al. (2020)
**Sterols:**					
**Campesterol**	Sunflower seed oil	ATCC 201249	Δ*erg5* ↑*XlDHCR7*	453 mg/L (bioreactor)	Du et al. (2016)
**Campesterol**	Sunflower seed oil	ATCC 201249	Δ*erg5* ↑*DrDHCR7* ↑*POX2*	942 mg/L (bioreactor)	Zhang et al. (2017)
**Triterpenoids:**					
**Betulinic acid**	Glycerol	ATCC 201249	↑*tHMG1* ↑*SQS* ↑*AtLUP1* ↑*MtCYP716A12*-t*AtATR1*	26.53 mg/L (shake flask)	Sun et al. (2019)
**Betulinic acid**	Glucose	ATCC 201249	↑*RcLUS* ↑*BPLO* ↑*LjCPR* ↑*SQS* ↑*SQE* ↑*HMG* ↑*MFE1*	204.89 mg/L total triterpenoid (shake flask)	Jin et al. (2019)
**Ginsenoside K**	Glucose	ATCC 201249	↑*tHMG* ↑*ERG20* ↑*SQS* ↑*PgDS* ↑*PgPPDS-L-tAtATR1* ↑*PgUGT1*	161.8 mg/L (bioreactor)	Li et al. (2019)
**Lupeol**	Glucose Pyruvic acid	ATCC 201249	*↑RcLUS ↑HMG ↑SQS ↑SQE ↑OLE1 Δpah1 Δdgk1*	411.72 (shake flasks)	(J.-L. Zhang et al., 2020)
**Oleanolic acid**	Glucose	ATCC 201249	*↑tHMG ↑ERG20 ↑SQS ↑GgBAS ↑MtCYP716A12-L-tAtATR1*	540.7 mg/L (bioreactor)	(D. Li et al., 2020)
**2,3-oxidosqualene**	Glucose	ATCC 20460	*↑HMG ↑ERG12 ↓ERG7 ↑ACL1 ↑SeACS*^*L641P*^*↑IDI ↑ERG20 ↑SQS ↑SQE*	22 mg/L (deepwell plate)	Arnesen et al. (2020)
**Protopanaxadiol**	Xylose	ATCC 201249	*↑SsXR ↑SsXDH ↑XKS ↑PgDS ↑PgPPDS-L-AtATR1 ↑tHMG ↑ERG20 ↑SQS ↑TKL ↑TAL ↑TX Δpox1,2,3*	300.63 mg/L (bioreactor)	Wu et al. (2019)
**Squalene**	Glucose Citrate	Po1f	*↑HMG ↑ACL1 ↑SeACS*^*L641p*^	10 mg/gDCW (shake flask)	Huang et al. (2018)
**Squalene**	Glucose	Po1f	*↑carB ↑carRP ↑ERG8,10,12,13,19,20 ↑tHMG ↑IDI Δgut2 Δpox3,4,5,6*	531.6 mg/L	Gao et al. (2017b)
**Squalene**	Glucose	ATCC 20460	*↑HMG ↑ERG12 ↓ERG7 ↑ACL1 ↑ SeACS*^*L641P*^*↑IDI ↑ERG20 ↑SQS*	402.4 mg/L (deepwell plate)	Arnesen et al. (2020)
**Squalene**	Glucose	Po1g	*↑SQS ↑HMG ↑MnDH2*	502.7 mg/L (shake flask)	(H. Liu et al., 2020)
**Squalene**	Glucose	Po1f	*Δpex10 Δure2 ↑HMG ↑DGA1*	240.5 mg/L (shake flask)	Wei et al. (2021a)
**Diterpenoids:**					
**Gibberellins**	Glucose	GB20	*↑tHMG ↑GGPPS ↓SQS ↑AtCPS ↑AtKS ↑AtKO ↑YlCyb5 ↑AtATR2 ↑SsGGPPS ↑GfCyb ↑GfCybRed ↑GfCPR ↑GfDES ↑GfP450-1 ↑GfP450-2 ↑GfP450-3 ↑tAtCPS ↑tAtKS ↑tAtKO*	12.81 mg/L GA_3_, 16.41 mg/L GA_4_, 0.79 mg/L GA_7_, and 4.70 mg/L GA_9_.	Kildegaard et al. (2021)
**Tetraterpenoids:**					
**Astaxanthin**	Glucose	GB20	*↑XdcrtYB ↑XdcrtI ↑HMG ↓SQS ↑XdcrtE ↑PscrtW ↑PacrtZ*	54.6 mg/L (microtiterplate)	Kildegaard et al. (2017)
**Astaxanthin**	Glucose	GB20	*↑XdcrtYB ↑XdcrtI ↑HMG ↓SQS ↑XdcrtE ↑SsGGPPS ↑HpBKT ↑HpCrtZ*	285 mg/L (bioreactor)	Tramontin et al. (2019)
**Astaxanthin**	Safflower oil	GB20	*↑XdcrtYB ↑XdcrtI ↑HMG ↓SQS ↑XdcrtE ↑PscrtW ↑PacrtZ*	167 mg/L (bioreactor)	(N. Li et al., 2020)
**Astaxanthin**	Glucose	Not described	*↑PsCrtW-HpCrtZ-SKL ↑PsCrtW-HpCrtZ-oleosin ↑PsCrtW-HpCrtZ-KDEL ↑SaGGPPS ↑McCarRP ↑McCarB*	858 mg/L (shake flasks)	(Yongshuo Ma et al., 2021)
**Cantaxanthin**	glucose	Po1f	*↑BsCrtW ↑McCarB ↑McCarRP ↑GGPPS*	36.1 mg/L (shake flask)	Cui et al. (2021)
**β-carotene**	Glucose	Po1f	*↑McCarB ↑McCarRP ↑ERG8,10,12, 13,19,20 ↑GGPPS ↑tHMG ↑IDI Δpox3,4,5,6*	4 g/L (bioreactor)	Gao et al. (2017b)
**β-carotene**	Glucose	Po1f	*↑GGPPS ↑SsCarS*	0.41 mg/gDCW	Gao et al. (2017a)
**β-carotene**	Glucose	ATCC 20460	*↑McCarB ↑McCarRP ↑HMG ↑GGPPS ↑DGA2 ↑GPD1 Δpox1–6 Δtgl4*	6.5 g/L (bioreactor)	Larroude et al. (2018)
**β-carotene**	Glucose	Po1g	*↑EcAtoB ↑ScERG13, ↑HMG ↑ERG8,12,19,20 ↑IDI ↑GGPPS ↑McCarB ↑McCarRP*	12.1 mg/gDCW	Cui et al. (2019)
**β-carotene**	Glucose	S11073 (in-house strain collection)	*↑McCarB ↑McCarRP*	75 mg/L (shake flask)	Bruder et al. (2020)
**β-carotene**	Glucose	ATCC 20460	*↑HMG ↑ERG12 ↑ACL1 ↑ SeACS*^*L641P*^*↑IDI ↑GGPPS ↓SQS ↑XdcrtYB ↑XdcrtI*	164 mg/L (deepwell plate)	Arnesen et al. (2020)
**β-carotene**	Glucose	Po1f	*↑McCarB ↑McCarRP ↑GGPPS ↑HMG ↑ERG13 Δpox2,3 Δmfe*	4.5 g/L (bioreactor)	(X.-K. Zhang et al., 2020)
**β-carotene**	Glucose	Po1f	*↑tHMG ↑BtCarB ↑BtCarRA ↑GGPPS Δgut2 ↑ERG13 ↑Hxk*	2.4 g/L (bioreactor)	Qiang et al. (2020)
**β-carotene**	Glucose	Po1f	*↑tHMG ↑CarB ↑CarRP ↑GGPPS Δgut2 ↑DID2*	2.01 g/L (bioreactor)	Lv et al. (2020)
**β-carotene**	Glucose	IMUFRJ 50682	*↑McCarB ↑McCarRP ↑GGPPS*	50.1 mg/L (shake flask)	de Souza et al. (2020)
**β-carotene**	Glucose	Po1f	*↑tHMG ↑BtCarB ↑BtCarRA ↑GGPPS Δgut2 ↑DID2*	∼2.6 g/L (bioreactor)	Yang et al. (2021)
**β-carotene**	Glucose	Po1f	*↑tHMG ↑GGPPS ↑BtCarRA ↑BtCarB*	1.7 g/L (bioreactor)	(L. Liu et al., 2021)
**β-carotene**	Glucose	Po1f	*↑McCarB ↑McCarRP Δndt80*	∼12.5 mg/gDCW (96-well plate)	Liu et al. (2022)
**β-carotene**	Glucose	Po1f	*ΔCLA4 ΔMHY1 ↑AfGGPS ↑IDI ↑ERG8,10,12,19,20 ↑VHb ↑MccarRP ↑GGPPS ↑McCarB ↑ScERG13 ↑HMG*	7.6 g/L (bioreactor)	(M. Liu et al., 2021)
**β-carotene**	Glucose	Po1f	*↑ScCK ↑AtIPK ↑ERG12 ↑tHMG ↑ERG20 ↑IDI ↑XdcrtE ↑McCarB ↑McCarRP*^*Y27R*^*or McCarRP*^*E78K*^	39.5 g/L β-carotene or 17.6 g/L lycopene, respectively (bioreactor)	Ma et al. (2022)
**Lycopene**					
**Lycopene**	Glucose Fructose	Po1f	↑*PaCrtE* ↑*PaCrtB* ↑*PaCrtI*	242 mg/L (bioreactor)	Nambou et al. (2015)
**Lycopene**	Glucose	H222	*↑PaCrtB ↑PaCrtI ↑GGPPS ↑HMG Δpox1–6 Δgut2*	16 mg/gDCW (bioreactor)	Matthäus et al. (2014)
**Lycopene**	Glucose	Po1f	*↑HMG ↑PaCrtE ↑PaCrtB ↑PaCrtI ↑ERG8,19*	213 mg/L (bioreactor)	Schwartz et al. (2017)
**Lycopene**	Glucose	Po1f	↑*PaCrtE* ↑*PaCrtB* ↑*PaCrtI* ↑AMPD	745 mg/L (bioreactor)	Zhang et al. (2019)
**Lycopene**	Glucose Palmitic acid	Po1f	*↑PvIDI ↑LpCrtE ↑LpCrtB ↑LpCrtI ↑AtIPK ↑ScCHK ↑ERG20*	4.2 g/L (bioreactor)	Luo et al. (2020)
**Apocarotenoids:**					
**α-ionone**	Glucose	Po1f	Proprietary information	408 mg/L (bioreactor)	Czajka et al. (2020)
**β-ionone**	Glucose	Po1f	↑Mc*CarB* ↑Mc*CarRP* ↑*OfCCD1* ↑*SsNphT7* ↑*HpIDI* ↑*ERG8,10,12,13,19* ↑*tHMG* ↑*GPPS* ↑*ERG20*-*GGPPS*	380 mg/L (bioreactor)	Czajka et al. (2018)
**β-ionone**	Glucose	Po1f	↑Mc*CarB* ↑M*cCarRP* ↑*PhCCD1* ↑*GGPPS* ↑*tHMG* ↑*ERG8,10,12,13,19,20* Δ*pox3,5* ↑*IDI* ↑*bbPK* ↑*bsPTA*	0.98 g/L (bioreactor)	Lu et al. (2020)

***AaADS***, *Artimisia annua* amorphadiene synthase. ***AaBFS***, *A. annua* β-farnesene synthase. ***AaLIS***, *Actinidia arguta* linalool synthase. ***AcACHS2***, *Aquilaria crassna* α-humulene synthase. ***ACL1***, ATP citrate lyase 1. ***AfGGPS***, *Archaeoglobus fulgidus* geranylgeranyl diphosphate synthase. ***AgαBS***, *Abies grandis* α-bisabolene synthase. ***AMPD***, adenosine monophosphate deaminase. ***ANT1***, peroxisomal adenine nucleotide transporter. ***AtATR1***, *Arabidopsis thaliana* cytochrome P450 reductase 1. ***AtATR2***, *A. Thaliana* cytochrome P450 reductase 2. ***AtCPS***, *A*. *Thaliana* copalyl diphosphate synthase. ***AtDTX***, A*. thaliana* abscisic acid exporter. ***AtIPK***, *A. thaliana* isopentenyl phosphate kinase. ***AtKO***, *A. Thaliana ent*-kaurene oxidase. ***AtKS***, *A*. *Thaliana ent*-kaurene synthase. ***AtLUP1***, *A*. *thaliana* lupeol synthase. ***BbPK***, *Bifidobacterium bifidum* phosphoketolase. ***BcABA1***, *Botrytis cinerea* cytochrome P450. ***BcABA2***, *B*. *cinerea* cytochrome P450. ***BcABA3***, *B*. *cinerea* α-ionylideneethane synthase. ***BcABA4***, *B*. *cinerea* dehydrogenase. ***BcCPR1***, *B*. *cinerea* cytochrome P450 reductase. ***BpHMG***, *Bordetella petrii* 3-Hydroxy-3-methylglutaryl-CoA reductase. ***BpLO***, *Betula platyphylla* lupeol C-28 oxidase. ***BsCrtW***, *Brevundimonas* sp. β-carotene ketolase. ***BsPTA***, *Bacillus subtilis* phosphotransacetylase. ***BtCarB***, *Blakeslea trispora* phytoene dehydrogenase. ***BtCarRA***, *B. trispora* phytoene synthase/lycopene cyclase. ***CarB****,* phytoene dehydrogenase from unknown species*.****CarRP****,* phytoene synthase/lycopene cyclase from unknown species*.****CLA4***, protein kinase involved in hyphal development. ***ClLS***, *Citrus limon* D-limonene synthase. ***ClSTS***, *Clausena lansium* α-santalene synthase. ***CnCYP706M1***-***AtATR1***, fusion of *Callitropsis nootkatensis* cytochrome P450 and *AtATR1*. ***CnVS***, *C*. *nootkatensis* valencene synthase. ***Cyb5***, Cytochrome *b*5. ***DGA1-2***, diacylglycerol acyltransferase 1–2. ***DGK1***, diacylglycerol kinase. ***DID2***, endosomal sorting complex subunit. ***DrDHCR1***, *Danio rerio* 7-dehydrocholesterol reductase. ***EcAtoB***, *Escherichia coli* acetyl-CoA acetyltransferase. ***ERG10***, acetyl-CoA acetyltransferase. ***ERG12***, mevalonate kinase. ***ERG13***, 3-hydroxy-3-methylglutaryl-CoA synthase. ***ERG19***, mevalonate diphosphate decarboxylase. ***ERG20***, farnesyl diphosphate synthase. ***ERG20***^***F88W***^–^***N119W***^, geranyl diphosphate synthase. ***ERG20***-***GGPPS***, fusion of ERG20p and geranylgeranyl diphosphate synthase. ***ERG5***, C22-sterol desaturase. ***ERG7***, lanosterol synthase. ***ERG8***, phosphomevalonate kinase. *FS-L-ERG20*, α-farnesene synthase from unknown species fused to ERG20p. ***GcABCG1***, *Grosmania clavigera* ABC-G1 efflux pump. ***GfCPR***, *Fusarium* (*Gibberella*) *fujikuroi* cytochrome P450 reductase. ***GfCyb***, *F*. *fujikuroi* cytochrome *b*5. ***GfCybRed***, *F*. *fujikuroi* cytochrome *b*5 reductase. ***GfDES***, F*. fujikuroi* desaturase. ***GfP450*-*1***, *F. fujikuroi* cytochrome P450 1. ***GfP450****-****2***, *F. fujikuroi* cytochrome P450 2. ***GfP450***-***3***, *F*. *fujikuroi* cytochrome P450 3. ***GgBAS***, *Glycyrrhiza glabra* β-amyrin synthase. ***GGPPS***, geranylgeranyl diphosphate synthase. ***GPD1***, glyceraldehyde-3-phosphate dehydrogenase. ***GPPS***, geranyl diphosphate synthase. ***GUT2***, glycerol-3-phosphate dehydrogenase 2. ***HaγBS***, *Helianthus annuus* γ-bisabolene synthase. ***HMG***, 3-Hydroxy-3-methylglutaryl-CoA reductase. ***HpBKT***, *Haematococcus pluvialis* β-carotene ketolase. ***HpCrtZ***, *H. pluvialis* β-carotene hydroxylase. ***HpIPI***, *H. pluvialis* isopentyl diphosphate isomerase. ***Hxk***, hexokinase. ***IDI***, isopentyl diphosphate isomerase. ***LjCPR***, *Lotus japonicus* cytochrome P450 reductase. ***LpCrtB***, *Lamprocystis purpurea* phytoene synthase. ***LpCrtE***, *L*. *purpurea* geranylgeranyl diphosphate syntase. ***LpCrtI***, *L*. *purpurea* phytoene desaturase. ***MBP***-***ERG12***, Maltose binding protein N-terminally fused to ERG12p. ***McCarB***, *Mucor circinelloides* phytoene dehydrogenase. ***McCarRP***, *M. circinelloides* phytoene synthase/lycopene cyclase. ***MdFS***-***L****-****ERG20***, *Malus domestica* α-farnesene synthase linked to ERG20p. ***MFE1***, multifunctional β-oxidation enzyme 1. ***MHY1***, transcription factor involved in hyphal formation. ***MnDH2***, Mannitol dehydrogenase. ***MrBBS***, *Matricaria recutita* (−)-α-bisabolol synthase. ***MsLS***, *Mentha spicata* L-limonene synthase. ***MtCYP716A12***-***L****-****tAtATR1***, *Medicago truncatula* cytochrome P450 fused to truncated AtATR1p. ***MtCYP716A12***-***tAtATR1***, *M*. *truncatula* cytochrome P450 directly linked to truncated AtATR1p. ***NDT80***, transcription factor affecting lipid and ergosterol biosynthesis. ***OfCCD1***, *Osmanthus fragrans* carotenoid cleavage dioxygenase 1. ***OLE1***, Δ9-fatty acid desaturase. ***PaCrtB***, *Pantoea ananatis* phytoene synthase. ***PaCrtE***, *P*. *ananatis* geranylgeranyl diphosphate syntase. ***PaCrtI***, *P*. *ananatis* phytoene desaturase. ***PacrtZ***, *P*. *ananatis* β-carotene hydroxylase. ***PAH1***, phosphatidic acid phosphatase. ***PfLS***, *Perilla frutescens* limonene synthase. ***PgDS***, *Panax ginseng* dammarenediol II synthase. ***PgPPDS***, *P. ginseng* cytochrome P450 enzyme. ***PgPPDS***-***L***-***AtATR1***, PgPPDSp linked to AtATR1p. ***PgPPDS***-***L****-****tAtATR1***, PgPPDSp linked to truncated AtATR1p. ***PgUGT1***, *P*. *ginseng* UDP-glycosyltransferase. ***PhCCD1***, *Petunia hybrida* carotenoid cleavage dioxygenase. ***PmISPS***, *Pueraria montana* isoprene synthase. ***POS5***, Putative NAD + kinase. ***POT1***, 3-ketoacyl-CoA thiolase. ***POX1-6***, peroxisome acyl-CoA oxidase 1–6. ***PsCrtW***, *Paracoccus* sp. β-carotene ketolase. ***PsCrtW*-*HpCrtZ*-*KDEL***, fusion of PsCrtW-HpCrtZp with endoplasmic reticulum targeting sequence. ***PsCrtW*-*HpCrtZ*-*oleosin***, fusion of PsCrtW-HpCrtZp with lipid body targeting signal. ***PsCrtW*-*HpCrtZ*-*SKL***, fusion of PsCrtW-HpCrtZp with peroxisome targeting signal. _***PTS***_, C-terminal peroxisomal targeting signal. ***PvIDI***, *Pseudescherichia vulneris* isopentyl diphosphate isomerase. ***RcLUS***, *Ricinus communis* lupeol synthase. ***RpHMG1***, *Ruegeria Pomeroyi* NADH-dependent HMGp. ***SaGGPPS***, *Sulfolobus acidocaldarius* geranylgeranyl diphosphate synthase. ***ScCHK***, *Saccharomyces cerevisiae* choline kinase. ***tScHMG***, truncated *S. cerevisiae* HMGp. ***SeACS***^***L641P***^, mutated *Salmonella enterica* acetyl-CoA synthetase. ***SQE***, squalene epoxidase. ***SQS***, squalene synthase. ***SsCarS***, Multifunctional *Schizochytrium* sp. carotene synthase. ***SsGGPPS***, *Synechococcus* sp. GGPPS. ***SsNphT7***, *Streptomyces* sp. acetoacetyl CoA synthase. ***SsXDH***, *Scheffersomyces stipites* xylose dehydrogenase. ***SsXR***, *S*. *stipites* xylose reductase. ***TAL***, transaldolase. ***tArLS***, truncated *Agastache rugosa* limonene synthase. ***tAtCPS***, truncated AtCPSp with plastidial targeting sequence removed. ***tAtKO***, truncated AtKOp with plastidial targeting sequence removed. ***tAtKS***, truncated AtKSp with plastidial targeting sequence removed. ***TGL3-4***, triacylglycerol lipase 3–4. ***TKL***, transketolase. ***tPtPS***, truncated *Pinus taeda* α-pinene synthase without plastidial targeting sequence. ***tSlNDPS1***, truncated *Solanum lycopersicum* neryl diphosphate synthase 1 without plastidial targeting sequence. ***TX***, xylose transporter. *URE2*, transcriptional regulator involved in nitrogen catabolism repression. ***VHb***, *Vitreoscilla stercoraria* hemoglobin. ***XdcrtE***, *Xanthophyllomyces* dendrorhous geranylgeranyl diphosphate synthase. ***XdcrtI***, *X. dendrorhous* phytoene desaturase. ***XdcrtYB***, *X. dendrorhous* bi-functional phytoene synthase/lycopene cyclase. ***XKS***, xylulose kinase. ***XlDHCR7***, *Xenapus laevis* 7-dehydrocholesterol reductase. ***ZoβBS***, *Zingiber officinal* β-bisabolene synthase. The table and accompanying text is expanded from (Arnesen et al., 2020).

## 3 Mevalonate pathway engineering

Engineering of the mevalonate pathway for terpenoid overproduction often involves the upregulation of MVA-pathway genes. In particular, the reduction of HMG-CoA to mevalonate catalyzed by HMGp is a popular target for upregulation (Ashour et al., 2010; Polakowski et al., 1998). This tendency is highlighted by the preponderance of the surveyed literature, which uses HMGp-upregulation as a terpenoid overproduction strategy (Table 1). The model yeast *S. cerevisiae* contains two *HMG*-genes encoding ScHMG1p and ScHMG2p, respectively (Burg and Espenshade, 2011). Both enzymes are regulated on the transcriptional level, but ScHMG1p is also regulated during translation, while ScHMG2p is post-translationally regulated by ubiquitination and endoplasmic reticulum-associated degradation. The negative feedback regulation of ScHMG1p can be overcome by truncating the membrane-anchored N-terminal (tScHMG1p), resulting in higher HMGp-activity (Polakowski et al., 1998). This led to the hypothesis that the same principle applies to *Y. lipolytica* HMGp, but several studies show that non-truncated HMGp outperforms tHMGp for terpenoid production in *Y. lipolytica* (Cao et al., 2016; Huang et al., 2018; Jia et al., 2019; Kildegaard et al., 2017; H. Liu et al., 2020; S. C. Liu et al., 2020), while a few other studies do not point to large differences (D. Li et al., 2020; Li et al., 2019). While some studies utilized heterologous NADH-dependent HMGp enzymes based on a presumed large abundance of NADH in *Y. lipolytica*, they did not compare these enzymes to overexpression of NADPH-dependent HMGp versions (Guo et al., 2021; Y. Liu et al., 2019). Therefore, it remains unclear whether shifting HMGp dependency from NAPDH to NADH is a superior strategy for terpenoid production in *Y. lipolytica*.

Besides HMGp upregulation, some studies demonstrate the benefits of overexpressing single or multiple MVA-genes for terpenoid production in *Y. lipolytica*. However, these modifications are typically co-overexpressed with HMGp, due to the long track record of this strategy for boosting terpenoid production. Two studies found that overexpression of ERG12p increased limonene yield (mass of product/dry biomass) 6-fold and seemingly also α-pinene titers (product concentration in cultivation broth) (Cao et al., 2016; Wei et al., 2021b). Conversely, another study showed no significant amorphadiene titers increase when ERG12p was overexpressed (Marsafari and Xu, 2020). Overexpression of ERG19p increased lycopene yield, and α-farnesene titer and yield in different studies (S. C. Liu et al., 2020; Schwartz et al., 2017). Overexpression of ERG13p also improved limonene titers by ∼20% in one study and β-carotene yield in two studies (Li et al., 2022; Qiang et al., 2020; X.-K. Zhang et al., 2020). Overexpression of IDIp increased α-farnesene titers in a tHMGp-expressing background, but not without tHMGp-expression (Yang et al., 2016). IDIp overexpression also increased linalool titers 2.8-fold in an HMGp-overexpressing background (Cao et al., 2017). Interestingly, three-copy, but not single-, or double-copy, overexpression of IDI benefitted linalool titers, but not limonene yield, without HMG-overexpression (Cao et al., 2016, 2017). This suggests that HMGp-upregulation influences the effects of other MVA-pathway modifications on terpenoid production. Another strategy is to upregulate the entire MVA-pathway. It was found that overexpression of all MVA-pathway genes (*ERG10,13,12,8,19,20*, *tHMG*, and *IDI*) boosted β-carotene production by 46% and β-ionone titers by 2.8-fold in separate studies, with the caveat that non-neutral genomic loci like *POX* genes were targeted for DNA construct integration (Gao et al., 2017b; Lu et al., 2020).

Furthermore, increasing the conversion of DMAPP/IPP into the appropriate terpene precursor GPP, FPP, or GGPP can be advantageous. The expression of a mutated farnesyl diphosphate synthase (ERG20^F88W^–^N119W^p) resulted in 0.56 mg/L linalool while the parental strain produced 0.09 mg/L (Cao et al., 2017). This strategy was based on a previous study in *S. cerevisiae* that demonstrated that the mutation of similar residues in *S. cerevisiae* ERG20p changed its function into a geranyl diphosphate synthase (Ignea et al., 2014). ERG20p overexpression resulted in 54.68 mg/L of the sesquiterpenoid (−)-α-bisabolol, while the parental strain only produced 39.83 mg/L (Yirong Ma et al., 2021). Likewise, overexpression of ERG20p alongside *S. cerevisiae* tHMGp-expression tScHMGp resulted in 22.8 mg/L (+)-valencene and 978.2 μg/L (+)-nootkatone, whereas only expressing tScHMGp provided 10.9 mg/L (+)-valencene and 551.1 μg/L (+)-nootkatone (Guo et al., 2018). Increasing the copy number of *ERG20* from two to three copies increased abscisic acid production with a strain-dependency (Arnesen et al., 2022).

The overexpression of GGPPSp resulted in a 4-fold increase in β-carotene titer (Kildegaard et al., 2017). In another study, the expression of the archaeal *Archaeoglobus fulgidus* GGPPS increased carotenoid yield 2.6-fold, while the combined overexpression of ERG20p and native GGPPSp only increased carotenoid yield by 1.9-fold (M. Liu et al., 2021). Likewise, expression of *Synechococcus* sp. (cyanobacterium) (SsGGPPS7p) increased β-carotene titers by 272%, while expression of a second *Xanthophyllomyces dendrorhous* GGPP synthase (*XdcrtE)* copy increased β-carotene titers by 49% (Tramontin et al., 2019). Interestingly, a comparison of GGPP productivity of GGPPSp/crtEp enzymes from various organisms put them in the order of *Taxus canadensis*, *Pantoea agglomerans*, *Y. lipolytica*, *Sulfolobus acidocaldarius*, and *X*. *dendrorhous* from lowest to highest (Ma et al., 2022). Interestingly, the expression of *S. acidocaldarius* GGPPSp (SaGGPPSp) provided the highest β-carotene titer and β-carotene to lycopene ratio compared to the other GGPPS enzymes. This was explained by the slightly lower flux of SaGGPPSp, avoiding too rapid lycopene build-up and therefore preventing substrate inhibition.

Another option for improving terpenoid production is to limit the flux of intermediates towards undesired side-products. In the case of non-triterpenoid or -sterol products, this may be achieved by reducing flux towards squalene. Replacement or truncation of the native *SQS* promoter was found to increase β-carotene titers by 2–2.5-fold (Kildegaard et al., 2017). Interestingly, some of these modifications also increased squalene titers. Contrarily, SQS-promoter replacement did not positively affect β-farnesene titer and yield (Arnesen et al., 2020). Promoter replacement of *SQS* with the glycerol repressible P_alk_-promoter resulted in decreased squalene yield and growth during cultivation on glycerol while increasing lycopene, but not total carotenoid, yield (Gao et al., 2017b). Truncation of the native *SQS* promoter slightly increased (−)-α-bisabolol titers and slightly decreased squalene titers (Yirong Ma et al., 2021).

In summary, engineering of the MVA-pathway is a well-established strategy for increasing terpenoid production in *Y. lipolytica,* although the effects do vary between the specific terpenoids and strains.

Another approach to increasing the supply of IPP in the cell is to enable conversion of isoprenol to IPP via two enzymatic steps. The cell culture is then co-fed with isoprenol, which provides plentiful amounts of IPP. Expression of the *Arabidopsis thaliana* isopentenyl phosphate kinase (AtIPKp) and *S. cerevisiae* choline kinase (ScCHKp) (so called Isopentenol Utilization Pathway IUP) increased the IPP/DMAPP-pool 15.7-fold in the PO1f background and lycopene yield in *Y. lipolytica* (Luo et al., 2020). Furthermore, introducing the IUP-pathway can enhance terpenoid biosynthesis complementary to MVA-pathway engineering. In a β-carotene producing strain overexpressing *tHMG*, *ERG12*, *IDI*, *ERG20*, and *XdcrtE,* the introduction of IUP increased β-carotene titers 23% (Ma et al., 2022).

## 4 Cofactor and acetyl-CoA engineering

Reducing HMG-CoA into mevalonate by HMGp in yeast requires NADPH as a cofactor (Polakowski et al., 1998). Therefore, a few studies have attempted to improve terpenoid production by increasing NADPH availability. The mannitol dehydrogenase makes NADPH from NADP+ during the conversion of mannitol into fructose (H. Liu et al., 2019). Yet, the overexpression of the mannitol dehydrogenase MnDH2p increased squalene titer slightly but did not benefit the yield (H. Liu et al., 2020). Another study found that triterpenoid titers increased somewhat in some strain backgrounds when malic enzymes, *Mortierella alpina* EMTp or *Rhodotorula toruloides* Rtmep, generate NADPH by decarboxylating malate into pyruvate and CO_2,_ were expressed (Jin et al., 2019). For campesterol production, overexpression of a malic enzyme decreased yield by 43% (Zhang et al., 2017). Overexpression of the putative NAD + kinase POS5p failed to increase abscisic acid production (Arnesen et al., 2022). Therefore, increasing NADPH availability currently seems to only offer small potential benefits for terpenoid production in *Y. lipolytica*, but more research on this topic is warranted.

**Fig. 2:**
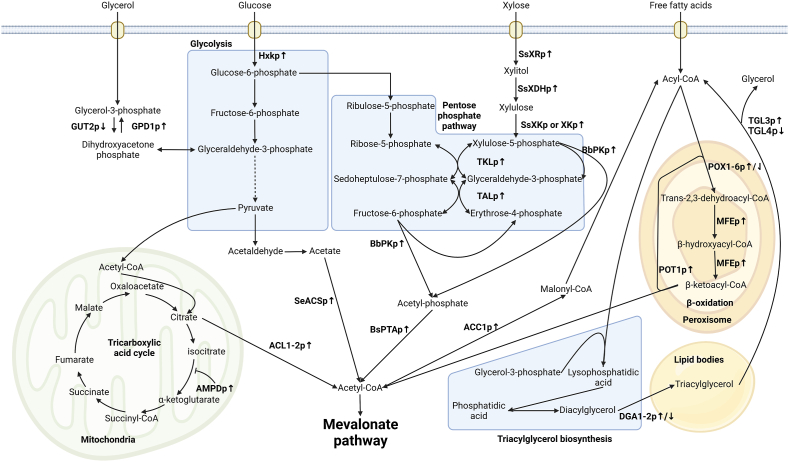
Overview of metabolic engineering strategies pertaining to acetyl-CoA, fatty acid metabolisms, and substrate utilization used in *Y. lipolytica* for terpenoid production in different studies. ↑, expressed or overexpressed. ↓, downregulated. Native enzymes: AMPDp, adenine monophosphate deaminase. GUT2p, glycerol-3-phosphate dehydrogenase 2. GPD1p, glyceraldehyde-3-phosphate dehydrogenase. Hxkp, Hexokinase. ACL1-2p, ATP-citrate lyases. ACC1p, acetyl-CoA carboxylase 1. TKLp, transketolase. TALp, transaldolase. XKp, xylulose kinase. POX1-6p, peroxisome acyl-CoA oxidases. MFEp, multifunctional β-oxidation enzyme 1. POT1p, 3-ketoacyl-CoA thiolase. DGA1-2p, diacylglycerol acyltransferases. TGL3-4p, triacylglycerol lipases. Non-native enzymes: SeACS^L641P^p, mutated *Salmonella enterica* acetyl-CoA synthetase. BbPKp, *Bifidobacterium bifidum* phosphoketolase. BsPTAp, *Bacillus subtilis* phosphotransacetylase. SsXRp, *Scheffersomyces stipites* xylose reductase. SsXDHp, *S. stipites* xylose dehydrogenase. SsXKp, *S. stipites* xylulose kinase.

*Y. lipolytica* is presumed to have high acetyl-CoA abundance relative to other common microbial chassis, which may have been inferred by the medium-to-high lipid accumulation obtained by some *Y. lipolytica* strains during particular cultivation conditions (Beopoulos et al., 2008; Kerkhoven et al., 2016). Some studies indicate that *Y. lipolytica* possesses higher acetyl-CoA flux and abundance than *S. cerevisiae* under similar cultivation conditions, but more evidence is needed to justify such generalized statements (Christen and Sauer, 2011; Dahlin et al., 2019). Nevertheless, strategies that increase acetyl-CoA flux and their effect on terpenoid production in *Y. lipolytica* have been studied (Fig. 2). The ATP-citrate lyase (ACLp) forms acetyl-CoA, oxaloacetate, and ADP + Pi from citrate and CoA in the cytosol (Blazeck et al., 2014; Dulermo et al., 2015). ACLp consists of two subunits, ACL1p and ACL2p, in *Y. lipolytica*. Overexpression of ACL1p with co-expression of a mutated version of the *Salmonella enterica* acetyl-CoA synthetase (SeACS^L641P^p), that forms acetyl-CoA, AMP, and diphosphate from acetate, CoA, and ATP, improved squalene yield by 3.2-fold (Huang et al., 2018). Individual expression of either SeACS^L641P^p or ACL1p did not significantly enhance squalene accumulation. Overexpression of SeACS^L641P^p and ACL1p also improved β-farnesene titer and yield (Arnesen et al., 2020). Furthermore, overexpression of ACL1p increased acetyl-CoA production in the PO1f background, while ACL2p-overexpression decreased acetyl-CoA accumulation (Huang et al., 2018). Curiously, the overexpression of ACL2p, but not ACL1p, increased the triterpenoid titers in some strain backgrounds, while both ACL1p and ACL2p overexpression increased lycopene production independently (Jin et al., 2019; Zhang et al., 2019). ACL2p overexpression also improved squalene titer, but decreased the yield due to a corresponding increase in biomass (H. Liu et al., 2020). ACLp overexpression increased campesterol yield 1.3-fold (Zhang et al., 2017). Overexpression of the adenine monophosphate deaminase (AMPDp) increased lycopene titer and yield (Zhang et al., 2019). AMPDp inhibit the isocitrate dehydrogenase, which increase citrate and, by extension, acetyl-CoA levels.

Expression of a non-native pathway for acetyl-CoA generation consisting of the *Bifidobacterium bifidum* phosphoketolase (BbPKp) and *Bacillus subtilis* phosphotransacetylase (BsPTA) increased β-ionone titer by 32% (Lu et al., 2020). The phosphoketolase can convert fructose-6-phosphate or xylose-5-phosphate into erythrose-4-phosphate or glyceraldehyde-3-phosphate, respectively, and acetyl-phosphate, while the latter can be converted into acetyl-CoA by the phosphotransacetylase (Bergman et al., 2016).

Alternatively, acetyl-CoA accumulation can be affected by decreasing flux towards lipid biogenesis or increasing rates of fatty acid degradation. Knocking out the diacylglycerol acyltransferase genes Δ*dga1* and Δ*dga2* reduced the lipid content from 26.3% to 8.7% and increased β-farnesene titers by 56.32% (T. Shi et al., 2021). Knocking out Δ*dga1* or Δ*dga2* separately seemingly provided lesser reductions in lipid content and lesser increases in β-farnesene titers. The peroxisome acyl-CoA oxidase 2 (POX2p) overexpression increased campesterol yield 1.3-fold (Zhang et al., 2017). The growth media contained sunflower oil with abundant long-chain fatty acids, likely providing a substrate for the enhanced β-oxidation pathway. Interestingly, overexpression of POX1,4,5,6p under the same conditions did not affect campesterol yield, while overexpression of POX3p, the multifunctional-oxidation protein (MFEp), and the peroxisomal oxoacyl thiolase (POTp) decreased campesterol yield. MFEp catalyzes hydration and dehydrogenation reactions during β-oxidation, while POT1p catalyzes the thiolytic cleavage of β-ketoacyl-CoA into acetyl-CoA and a shortened acyl-CoA molecule (Beopoulos et al., 2009; Hanko et al., 2018; Smith et al., 2000). Interestingly, overexpression of MFE1p and POT1p increased (−)-α-bisabolol titers (Yirong Ma et al., 2021). Overexpression of POT1p also enhanced α-humulene titer and yield in a strain where the MVA- and α-humulene biosynthetic pathways were targeted to the peroxisomes, while overexpression of MFE1p or the peroxisomal biogenesis factor 10 (PEX10p) did not provide substantial benefits (Guo et al., 2021). MFE1p and POT1p overexpression also increased triterpenoid titers in some strain backgrounds (Jin et al., 2019). Strain-dependent increases in triterpenoid titers were also found when the long-chain fatty acid transporter (PXA1p) or triacylglycerol lipase (TGL3p) were overexpressed. Alternatively to genetic engineering, adding the lipid biosynthesis blocking compound cerulenin to the growth media enhanced the amorphadiene titer by 231.13% with a strain-specific dependency (Marsafari and Xu, 2020). Lastly, expression of *Vitreoscilla* hemoglobin (Vhbp) was demonstrated to improve α-farnesene production by 12.7%, likely by enhancing oxygen delivery to the cells (Y. Liu et al., 2021).

## 5 Modulation of lipid storage

Some hydrophobic terpenoids like β-carotene are stored in the lipid bodies of *Y. lipolytica* (Larroude et al., 2018). Therefore, it may be beneficial to increase sequestration of such lipophilic products by expanding the lipid pool. Larroude et al. developed an obese *Y. lipolytica* platform strain by overexpression of *DGA2*, the glyceraldehyde-3-phosphate dehydrogenase gene (*GPD1*), and deletion of Δ*pox1-6* and Δ*tgl4* (Larroude et al., 2018). This obese strain accumulated 3.6-fold more lipids and the β-carotene titer and yield were boosted by 1.9- and 2.6-fold compared to the wt. Overexpression of *DGA1* increased squalene yield by 2.9-fold and seemingly increased lipid accumulation (Wei et al., 2021a). Likewise, deletion of Δ*pex10* increased squalene production 9-fold. Furthermore, the co-deletion of Δ*ure2*, which encodes a protein putatively involved in oxidative stress responses and nitrogen metabolism, and Δ*pex10* improved squalene production more than the individual deletions. Overexpression of *DGA1* and the acetyl-CoA carboxylase gene (*ACC1*) increased the lipid content by 2.97- or 1.76-fold depending on the strain background, increased the accumulation of GPP, FPP, and GGPP, and seemingly benefitting lycopene production (Luo et al., 2020). Furthermore, overexpression of either GPD1p, DGA1p, or DGA2p increased lycopene titer and yield (Zhang et al., 2019). Presumably, engineering strategies for increased fatty acid accumulation may be advantageous specifically for intracellularly accumulated products. Indeed, deletion of Δ*pex10* decreased the titer and yield by >50% of the extracellularly accumulated α-farnesene, possibly due to reduced acetyl-CoA availability (S. C. Liu et al., 2020).

The overexpression of the Δ9-fatty acid desaturase gene (*OLE1*) combined with the deletion of the diacylglycerol kinase (Δ*dgk1*) and phosphatidic acid phosphatase (Δ*pah1*) genes increased the lupeol titer 4.7-fold while skewing the fatty acid profile from dominantly saturated to unsaturated (J.-L. Zhang et al., 2020). These gene edits also increased the ratio of extracellularly to intracellularly accumulated lupeol during two-phase cultivations with an organic overlay and were shown to benefit the production of α- and β-amyrin, and longifolene-type sesquiterpenes. These results were primarily attributed to increased plasma membrane permeability following lipid unsaturation allowing increased efflux into the extracellular phase.

A β-carotene producing strain was used to construct a mutant library by NHEJ-mediated random mutagenesis (Liu et al., 2022). This approach identified four new gene targets that improved β-carotene when a leucine prototrophy conferring gene-cassette (*LEU2*) was integrated with proximity to or into the gene. The largest increase in β-carotene production was achieved by NHEJ-mediated insertion of *LEU2* into the *NDH80*-locus. Reverse engineering by deletion of Δ*NDT80* in the parental strain increased β-carotene production by 62%. Furthermore, the lipid content increased, while the ergosterol content decreased in the Δ*NDT80*-strain compared to the parental strain, which may simultaneously expand the β-carotene storage capabilities and redirect MVA-pathway flux towards carotenoid biosynthesis.

## 6 Compartmentalization, morphology, and transport engineering

Several studies have applied pathway compartmentalization for terpenoid production in yeast (Dusséaux et al., 2020; G. S. Liu et al., 2020; Y. Shi et al., 2021).

Targeting the peroxisomes for α-humulene biogenesis was achieved by fusing a peroxisomal targeting signal (PTSp) peptide to the MVA-pathway enzymes and the *Aquilaria crassna* α-humulene synthase (AcHS_PTS_p) (Guo et al., 2021). The report demonstrated that only complete, but not partial, peroxisomal re-construction of the MVA-pathway improved α-humulene titers (50-fold) together with AcHS_PTS_p-expression. Further increases in α-humulene production were achieved by β-oxidation engineering (see previous chapter) and overexpression of the peroxisomal adenine nucleotide transporter gene (*ANT1*). This latter strategy increased the α-humulene titer by 11%, likely due to increased transport of cytoplasmic ATP into the peroxisomes. While the cited work resulted in a highly productive α-humulene strain (3.2 g/L), it is unclear whether the peroxisomal targeting strategy was superior to conventional cytoplasmic expression. However, another report does provide some evidence that compartmentalization can be beneficial for terpenoid production in *Y. lipolytica*. Increasing the copy number of a non-targeted astaxanthin biosynthetic fusion enzyme gene, consisting of the *Paracoccus* sp. β-carotene ketolase fused to the N-terminal of the *Haematococcus pluvialis* β-carotene hydroxylase (*PsCrtW-HpCrtZ*), did not affect astaxanthin production (Yongshuo Ma et al., 2021). Yet, the astaxanthin titer increased 1.62- to 1.84-fold when PsCrtW-HpCrtZp was targeted to either the lipid bodies, ER, or peroxisomes by fusion with the appropriate signal peptides. When PsCrtW-HpCrtZp was simultaneously targeted to all three compartments, the astaxanthin titer increased 4.8-fold. Expression of either the *Escherichia coli* resistance-nodulation-cell division family efflux pump (EcAcrBp) or the *Grosmania clavigera* ATP-binding cassette transporter (GcABCG1p) increased the titers of various bisabolene isomers during two-phase organic cultivation (Zhao et al., 2021).

Deleting genes involved in the single cells to hyphae transition can improve terpenoid production (M. Liu et al., 2021). Deleting the protein kinase gene (*ΔCLA4*) involved in the transition from yeast cells to filaments increased the β-carotene yield by 81%, although pseudohyphae could still be observed. Likewise, the deletion of the transcription factor gene (*ΔMHY1*) abolished hyphae formation and increased the β-carotene yield by 45%. Overexpression of endosomal sorting complex subunit DID2p increased β-carotene yield by 260%. In addition, it increased the expression of specific genes involved in β-carotene biosynthesis, the pentose phosphate pathway, the tricarboxylic acid pathway, and the hexokinase encoding gene (*Hxk*) (Yang et al., 2021).

While the above examples provide exciting investigations into the topics of compartmentalization, morphology engineering, and cellular transport, these research areas remain relatively unexplored in *Y. lipolytica*. It would be exciting for future research to expand these areas, which show great promise.

## 7 Enzyme engineering

Some papers demonstrate the utility of engineered enzymes for terpenoid production in *Y. lipolytica*. A common pathway improvement strategy is fusing proteins catalyzing consecutive biocatalytic steps or linking enzymes with supporting protein partners.

The pairing of the *Paracoccus* sp. ketolase (PsCrtWp) and the *Haematococcus pluvialis* hydroxylase (HpCrtZp) provided the highest astaxanthin titer in a small combinatorial screen (Yongshuo Ma et al., 2021). Fusing PsCrtWp and HpCrtZp via a protein linker increased the astaxanthin titers compared to separate expression, with 2.2- or 2.8-fold increases for the HpCrtZ-linker-PsCrtWp or PsCrtW-linker-HpCrtZp fusion protein, respectively. Fusion of the *Malus x domestica* α-farnesene synthase (MdFSp) with ERG20p increased α-farnesene titers compared to separate expression (Yang et al., 2016). The MdFS-linker-ERG20p fusion outperformed the ERG20p-linker-MdFSp fusion by 30%. Direct fusion of the *Callitropsis nootkatensis* cytochrome P450 (CnCYP706M1p) with the N-terminally truncated *A. thaliana* cytochrome P450 reductase 1 (AtATR1p) increased (+)-nootkatone titers ∼6-fold compared to separate expression of CnCYP706M1p and non-truncated AtATR1p, although multi-loci integration was used for both constructs (Guo et al., 2018). Interestingly, adding a “GSTSSG”-linker between CnCYP706M1p and tAtATR1p seemingly reduced the (+)-nootkatone titer. Fusion of the protopanaxadiol synthase (PPDSp), a cytochrome P450, and AtATR1p also increased protopanaxadiol titers 2.3-fold compared to co-expression (Wu et al., 2019). Truncation of the AtATR1p N-terminal is likely necessary due to the presence of a transmembrane domain. Indeed, no benefits to triterpenoid production were found when non-truncated cytochromes P450 and reductases were fused with varying linker lengths or C-/N-terminal configurations (Jin et al., 2019). Other studies have also used cytochrome P450 and reductases fusions to produce oleanolic acid, betulinic acid, and ginsenoside compound K (D. Li et al., 2020; Li et al., 2019; Sun et al., 2019).

Protein tagging can improve stability and solubility. For example, expression of ERG12p N-terminally tagged with maltose-binding protein (MBP) increased α-pinene 1.84-fold compared to overexpression of the untagged ERG12p (Wei et al., 2021b).

The effects of protein fusion and removal of the plastidial targeting sequences by N-terminal truncation of the *A. thaliana* copalyl diphosphate synthase (AtCPSp), *ent*-kaurene synthase (AtKSp), *ent*-kaurene oxidase (AtKOp) on GA-biosynthesis was investigated (Kildegaard et al., 2021). The highest GA-titers were achieved by expressing the non-fused truncated enzymes.

Structure-guided protein engineering of the R domain/lycopene cyclase from McCarRPp successfully removed substrate inhibition by lycopene (Ma et al., 2022). Structure modeling and a Position-Specific Scoring Matrix (PSSM) based on the lycopene cyclase domain and its homologs provided an evolutionary basis for selecting amino acid residues for substitution. A sampling of the variance space allowed the isolation of three McCarRPp variants, V175W, T31R–F92W, and Y27R, with improved β-carotene to lycopene ratios. The Y27R-variant had a 98% selectivity compared to 18% for the WT for β-carotene.

## 8 Alternative substrate utilization

The broad substrate utilization of *Y. lipolytica* makes it an excellent chassis for turning unconventional low-value or waste carbon sources into high-value products. Although glucose is commonly used as a substrate for terpenoid production in *Y. lipolytica* (Table 1) in the lab, for industrial processes, it can be advantageous to use cheaper or more abundant feedstock. It was demonstrated that oleic acid could be used as a carbon source for α-farnesene production with the final titer of 10.2 g/L achieved during fed-batch cultivation (Y. Liu et al., 2021). Oleic acid seemed to provide slightly better titers compared to glucose. α-farnesene was also produced in the 1–2 g/L range when cultivated in shake flasks with soybean oil, either fresh or from as waste cooking oil (WCO), olive, palm, glycerol trioleate, or rapeseed oil. Likewise, substituting oleic acid for glucose at carbon equivalent concentrations increased α-humulene titers by 18.5% for a strain with the MVA- and α-humulene pathway targeted to the peroxisomes (Guo et al., 2021). Using safflower oil with an oleic acid content of 77.0% as a carbon source during fed-batch cultivation resulted in 167 mg/L astaxanthin with 48% accumulated extracellularly (N. Li et al., 2020). The production of astaxanthin per cmol of carbon when using safflower oil as a carbon source was similar to that of glucose. Using sunflower seed oil at as carbon source resulted in higher campesterol titers than glucose at carbon equivalent concentrations (Du et al., 2016). It was also observed that sunflower oil increased lipid accumulation and lipid body formation compared to glycerol or glucose. Limonene titers were higher with WCO than glycerol or glucose at carbon equivalent concentrations (Li et al., 2022). Conversely, bisabolene titers were lower when using WCO than glucose at carbon equivalent concentrations, despite higher biomass accumulation when using WCO (Zhao et al., 2021). The addition of Mg^2+^ to WCO-based rich media increased the bisabolene titers, which was also found for limonene production (Pang et al., 2019). It was demonstrated that WCO, sunflower, rapeseed, or soybean oil could be used for α-pinene production, with the latter noted to increase α-pinene titer 80% compared to glucose (Wei et al., 2021b). The expression of the xylose metabolism genes encoding the *Scheffersomyces stipites* xylitol reductase (*SsXR*), *S. stipites* xylitol dehydrogenase (*SsXDH*), and native xylulokinase (*XK*) enabled α-pinene production and highly enhanced growth on xylose-containing rich media. But the expression of the xylose metabolism genes did not benefit α-pinene production when detoxified lignocellulosic hydrolysate, with major constituents being glucose, xylose, and acetate, was used as carbon source. Another report demonstrated that expressing the genes encoding *SsXR*, *SsXDH*, and *S. stipites* xylulokinase (*SsXK*) or *XK* enabled growth on xylose-based rich media (Yao et al., 2020). The expression of *XK* yielding better growth than *SsXK*. A mixture of 8 g/L glucose and 32 g/L xylose provided higher limonene titers compared to 40 g/L of either pure glucose or xylose for the engineered strain. Likewise, it was demonstrated improved growth on xylose based media by expressing *SsXR*^*K270R/N272D*^, *SsXDH*, and *XK* (Wu et al., 2019). The ability of the strain to consume xylose was improved by an adaptation period on xylose containing media, after which further engineering enabled protopanaxadiol (PPD) production. Overexpression of the transketolase (TKLp) and transaldolase (TALp), which connect the xylose degradation pathway with the pentose phosphate pathway, improved the PPD titer and biomass accumulation on xylose based media. The final engineered strain exhibited the best PPD titers on pure xylose as a carbon source compared to glucose or mixed sugar compositions.

Using glycerol instead of glucose, soybean, corn oil, or oleic acid at the same concentrations resulted in the highest betulinic acid titer (Sun et al., 2019). Interestingly, the expression of specific MVA-pathway genes and acetyl-CoA accumulation increased during cultivation with glycerol compared to glucose. Another report found that glycerol improved limonene yield compared to glucose, citrate, fructose, maltose, sucrose, mannose, or galactose at similar concentrations (Cheng et al., 2019). Adding auxiliary carbon sources like citrate, pyruvate, malate, but not acetate increased limonene titers further. Similarly, α-farnesene titer and yield during fed-batch bioreactor cultivation also improved when glycerol was fed instead of glucose, although the batch phase used glucose as a carbon source (S. C. Liu et al., 2020). Contrarily, glycerol decreased limonene yield or α-pinene titer and yield compared to glucose at similar concentrations (Cao et al., 2016; Wei et al., 2021b).

Using citrate as a carbon source resulted in the highest linalool yield and titer compared to fructose, glucose, or glycerol, with the latter being the second-best carbon source (Cao et al., 2017). The addition of pyruvate further increased linalool titer and yield. Likewise, the addition of pyruvic acid also increased limonene yield (Cao et al., 2016). The addition of citrate or acetate increased squalene yield for a strain expressing ACL1p and SeACS^L641P^p, which respectively can convert these substrates to acetyl-CoA (Huang et al., 2018). The addition of 4 g/L citrate to YPD medium enhanced α-pinene yield and titer, which was not found for the addition of pyruvate, acetate, or malate in the range of 0–4 g/L, or lower concentrations of citrate (Wei et al., 2021b).

The ability of *Y. lipolytica* to utilize glucose was enhanced by overexpression of the hexokinase (HXKp), which catalyzes the phosphorylation of glucose as the initial step in the glycolysis pathway (Qiang et al., 2020). HXKp overexpression enhanced β-carotene yield by 98% and led to faster glucose consumption.

Using palmitic acid as an addition to glucose-based rich media enhanced lycopene titers, lipid accumulation, and the accumulation of MVA-pathway metabolites like FPP and GGPP (Luo et al., 2020). Experiments using C_13_-labeled glucose showed that >90% of C16:0 and C18:0 lipids were unlabeled and therefore most likely derived from the exogenous lipid source, while ∼75% acetyl-CoA was derived from the labeled glucose. These findings are supported by (Ma et al., 2022), that demonstrated abundant unlabeled IPP/DMAPP and GGPP during cultivation with labeled glucose and unlabeled stearic acid. Therefore, the addition of fatty acids could enhance intracellular terpenoid storage and contribute to the acetyl-CoA pool via β-oxidation.

In summary, carbon sources like glycerol or WCO can be used as substrates for terpenoid production in *Y. lipolytica*. Furthermore, the research shows that the substrate acceptance of *Y. lipolytica* can be widened by engineering. Various auxiliary carbon sources also show promise; but their utility may be decided by their price-to-benefit ratio. Therefore, terpenoid production by cultivating *Y. lipolytica* with alternative and cheap carbon sources represents a promising avenue for developing economic and sustainable bioprocesses.

## 9 Outlook

The current research has demonstrated numerous varied strategies for improving terpenoid production in *Y. lipolytica*. However, while some strategies achieve different results across studies, it is important to consider that the contexts vary greatly; factors like strain background, cultivation conditions, properties of the specific terpenoid product and pathway, and previous strain modifications potentially affect the outcomes. Nevertheless, direct MVA-pathway engineering has been shown in multiple studies to improve terpenoid titers several-fold. Cofactor engineering by redirecting flux towards cytosolic acetyl-CoA has also shown some utility. Conversely, increasing lipid accumulation for intracellular storage is highly effective for producing some terpenoids, although likely dependent on the product's propensity to accumulate intra- or extracellularly. Emerging evidence demonstrates new strategies like morphology or compartmentalization engineering that increase terpenoid production in *Y. lipolytica*. Still, more research is needed to identify in which contexts these strategies are effective. Besides modifying the native metabolism, the careful selection and engineering of heterologous pathway and supportive enzymes by protein fusion, tagging, or modifying select amino residues can lead to several-fold increased terpenoid titers in *Y. lipolytica*. Media optimization is a valuable addition to genetic engineering, and alternative substrates and additives have been used to modestly increase terpenoid production in some reports. In summary, there are ample well-described and nascent strategies for improving terpenoid production in *Y. lipolytica*, and highly productive *Y. lipolytica* cell factories for terpenoids with short heterologous biosynthetic pathways have been developed. Indeed, the implementation of long heterologous biosynthetic pathways in *Y. lipolytica* remains a challenge with a good example being the production of gibberellin plant hormones (Kildegaard et al., 2021). Before the high-level production of complex terpenoids becomes possible, greater knowledge about the expression of individual enzymes and balancing long non-native pathways in *Y. lipolytica* is needed. Computational approaches utilizing genome-scale modelling, omics, and machine learning have yet to substantially impact terpenoid production in *Y*. *lipolytica*. These tools may become more relevant as they develop and the knowledge of *Y. lipolytica* metabolism expands.

## Declaration of competing interest

The authors declare that they have no known competing financial interests or personal relationships that could have appeared to influence the work reported in this paper.
